# Poly[tetra­kis­(dimethyl­formamide)tris(μ_4_-terephthalato)trimagnesium]

**DOI:** 10.1107/S1600536812038949

**Published:** 2012-09-22

**Authors:** Vandavasi Koteswara Rao, Matthias Zeller, Sherri R. Lovelace-Cameron

**Affiliations:** a Department of Chemistry, Youngstown State University, One University Plaza, Youngstown, OH 44555, USA

## Abstract

The title framework compound, [Mg_3_(C_8_H_4_O_4_)_3_(C_3_H_7_NO)_4_]_*n*_ or [Mg_3_(bdc)_3_(DMF)_4_]_*n*_, was obtained as a side product of the solvothermal reaction of magnesium nitrate, terephthalic acid (bdcH_2_), and 1,3-bis­(4-pyrid­yl)propane in a 1:2:1 ratio in dimethyl­formamide (DMF). The asymmetric unit consists of three Mg^II^ cations, three terephthalate anions, and four coordinating DMF mol­ecules. One of the four DMF mol­ecules was refined as disordered over two mutually exclusive positions, with an occupancy rate for the major moiety of 0.923 (4). The three Mg^II^ cations possess distorted octa­hedral coordination geometries that form linear Mg trimers. Of the three Mg^II^ cations, the central Mg^II^ is octa­hedrally coordinated by six different carboxyl­ate O atoms. The terminal Mg^II^ cations are bonded to four O atoms of three bdc linkers and to two O atoms of coordinating DMF mol­ecules. The compound has a two-dimensional 3^6^-network structure parallel to (001) that is formed by connection of the Mg trimers as distorted octa­hedral nodes to the bdc ligands as linkers.

## Related literature

For background information on Mg- and Zn-bdc metal-organic frameworks, see: Mallick *et al.* (2011 ▶); Burrows *et al.* (2005 ▶); Edgar *et al.* (2001 ▶); Grzesiak *et al.* (2006 ▶); Rood *et al.* (2006 ▶); Davies *et al.* (2007 ▶); Williams *et al.* (2005 ▶).
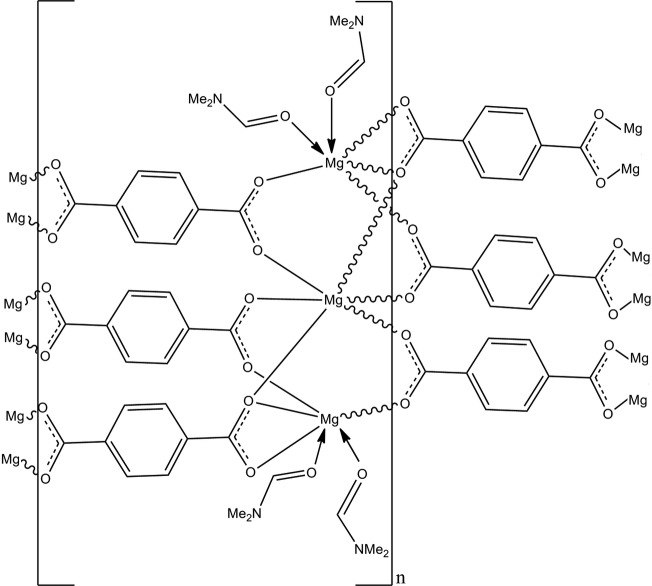


## Experimental

### 

#### Crystal data

[Mg_3_(C_8_H_4_O_4_)_3_(C_3_H_7_NO)_4_]
*M* *_r_* = 857.65
Monoclinic, 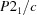

*a* = 18.158 (2) Å
*b* = 9.5046 (13) Å
*c* = 24.066 (3) Å
β = 100.825 (2)°
*V* = 4079.6 (10) Å^3^
*Z* = 4
Mo *K*α radiation
μ = 0.15 mm^−1^
*T* = 100 K
0.34 × 0.20 × 0.05 mm

#### Data collection

Bruker SMART APEX CCD diffractometer
Absorption correction: multi-scan (*SADABS*; Bruker, 2011 ▶) *T*_min_ = 0.619, *T*_max_ = 0.746
21816 measured reflections
10014 independent reflections
7598 reflections with *I* > 2σ(*I*)
*R*_int_ = 0.039

#### Refinement

*R*[*F* ^2^ > 2σ(*F* ^2^)] = 0.066
*wR*(*F* ^2^) = 0.149
*S* = 1.12
10014 reflections
574 parameters
33 restraints
H-atom parameters constrained
Δρ_max_ = 0.46 e Å^−3^
Δρ_min_ = −0.35 e Å^−3^

### 

Data collection: *APEX2* (Bruker, 2011 ▶); cell refinement: *SAINT* (Bruker, 2011 ▶); data reduction: *SAINT*; program(s) used to solve structure: *SHELXTL* (Sheldrick, 2008 ▶); program(s) used to refine structure: *SHELXLE* (Hübschle *et al.*, 2011 ▶) and *SHELXL97* (Sheldrick, 2008 ▶); molecular graphics: *DIAMOND* (Brandenburg, 2001 ▶); software used to prepare material for publication: *publCIF* (Westrip, 2010 ▶).

## Supplementary Material

Crystal structure: contains datablock(s) I, global. DOI: 10.1107/S1600536812038949/tk5144sup1.cif

Structure factors: contains datablock(s) I. DOI: 10.1107/S1600536812038949/tk5144Isup2.hkl

Additional supplementary materials:  crystallographic information; 3D view; checkCIF report

## Figures and Tables

**Fig. 1.**
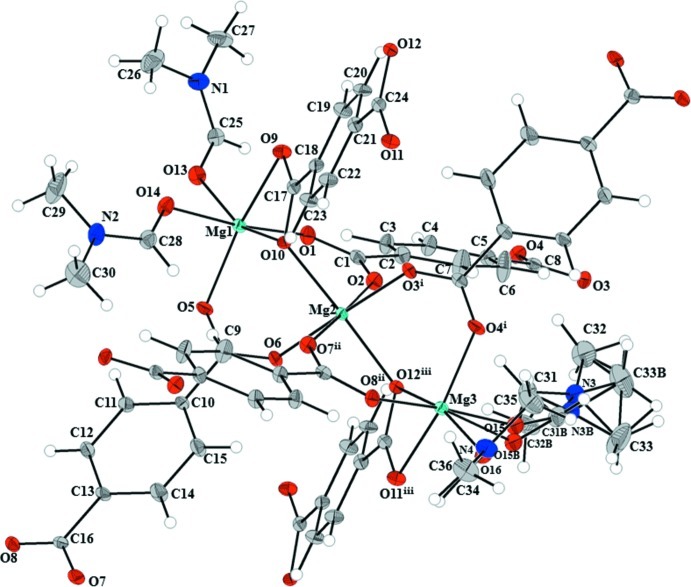
View of the title framework compound with the atom numbering scheme and 50% probability displacement ellipsoids. Symmetry operators: (i) -x + 1, y + 1/2, -z + 1/2; (ii) -x, y + 1/2, -z + 1/2; (iii) x, y - 1, z.

**Fig. 2.**
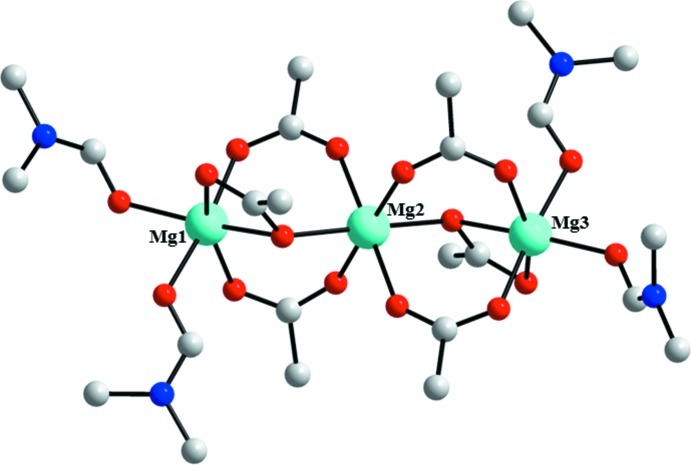
View of one trimeric SBU that acts as a node, possessing bridging carboxylates and terminal DMF molecules. For clarity, only the carboxylate groups of bdc linkers are shown and hydrogen atoms are omitted.

**Fig. 3.**
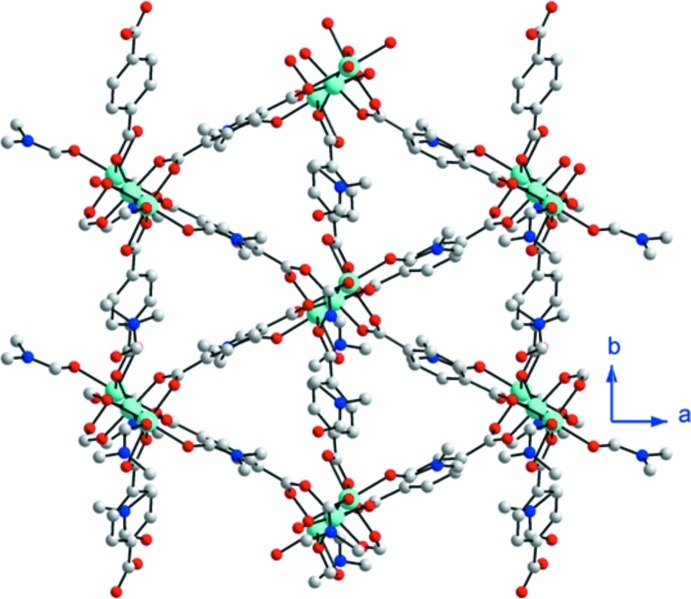
View of the two-dimensional 36-net parallel to the [001] direction. Hydrogen atoms are omitted for clarity.

**Fig. 4.**
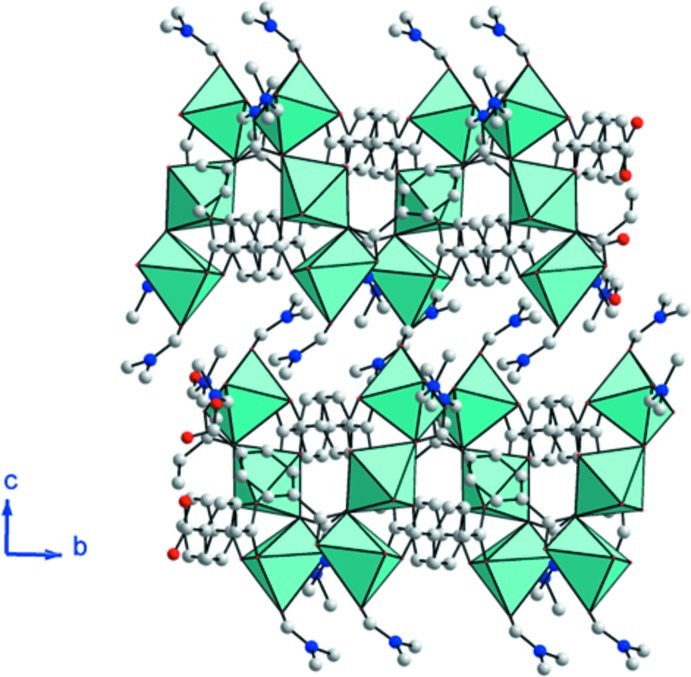
View of the arrangement of layers parallel to the [100] direction.

## supplementary crystallographic information

### Comment

The construction of metal-organic frameworks (MOFs) with light building blocks has been the subject of intense research during the past few years (Mallick *et al.*, 2011). A suitable metal for construction of lightweight frameworks is magnesium. In the present study, the title compound, [Mg_3_(bdc)_3_(DMF)_4_]_n_, was obtained during part of our investigations into the solvothermal synthesis of lightweight metal organic frameworks. Reaction of magnesium nitrate with terephthalic acid (bdcH_2_) and 1,3-bis(4-pyridyl)propane (tmdpyr) at 373 K (100 °C) in dimethyl formamide (DMF) yielded the title compound as a minor product along with an unidentified white powder. The crystals of the title compound are sensitive towards solvent loss and decompose rapidly once taken out of solution.

The asymmetric unit of the title framework consists of three magnesium cations, and three anionic bdc units, and four coordinated DMF molecules, respectively (Fig. 1). One of the four DMF molecules was refined as disordered over two mutually exclusive positions with an occupancy ratio of 0.923 (4) to 0.077 (4).

The three magnesium cations in the structure form secondary building units (SBUs) that each possesses a chain of three magnesium ions bridged by six bdc linkers and coordinated by four terminal DMF molecules (Fig. 2). There are two types of magnesium coordination environments in the structure. The terminal ions, Mg1 and Mg3, in the SBU exhibit distorted octahedral geometries. Of the six bonds, four are from O atoms of three different bdc linkers (one bdc linker binds with magnesium in a chelating mode) and the other two are from DMF O atoms. The central ion, Mg2, is also distorted octahedrally coordinated, but it binds to six different carboxylate O atoms (four of them are in bidentate mode and two are in chelation mode). O—Mg—O angles vary between 60.35 (7) and 178.54 (9)°, with the unusually small angle of 60.35 (7)° being associated with the bdc linker in chelation mode. The three distorted octahedral trimeric magnesiums together act as a node that is connected with other nodes through the bdc linkers to give rise to a two-dimensional layered 3^6^-network (Fig. 3). The layers in the structure are stacked atop of one another, with terminal DMF molecules that are projecting into the inter-lamellar space (Fig. 4). The structure is isoreticular to previously reported magnesium and bdc based framework compounds, Mg_3_(bdc)_3_(*X*)_4_ [*X* = DMSO, Rood *et al.*, 2006; DMA, Davies *et al.*, 2007] in which the terminal solvent molecules, DMSO and DMA, project in to the inter-lamellar spaces. Similar layered structures have also been reported with zinc-bdc based frameworks (Edgar *et al.*, 2001; Burrows *et al.*, 2005; Williams *et al.*, 2005; Grzesiak *et al.*, 2006).

### Experimental

The compound was synthesized under solvothermal conditions. In a typical synthesis, Mg(NO_3_)_2_.6H_2_O (0.129 g, 1.0 mmol) and terepthalic acid (0.169 g, 2.0 mmol) were dissolved in DMF (5.0 ml). Then, 1,3-bis(4-pyridyl)propane (0.101 g, 1.0 mmol) was added to the reaction mixture and stirred for one hour before transferring the mixture into a glass vial. The final mixture was heated to 373 K for 24 h. The vial was then slowly cooled to room temperature yielding colorless plates of the title compound as a minor product along with an unidentified white powder. Crystals are sensitive towards solvent loss and decompose rapidly once taken out of solution.

### Refinement

Reflections 1 1 0, 0 1 1 and 1 0 2 were partially obstructed by the beam stop and were omitted from the refinement.

One of the DMF molecules was refined as disordered over two mutually exclusive positions. The minor moiety was restrained to have a similar geometry as the major moiety, the overlapping O and N atoms were each constrained to have ADPs identical to that in the major moiety, and the ADPs of the C, N and O atoms of the minor moiety were restrained to be similar to each other. The occupancy ratios for the two moieties refined to 0.923 (4) to 0.077 (4).

Carbon-bound hydrogen atoms were placed in calculated positions with C—H bond distances of 0.95 Å (aromatic H and carbonyl H of DMF) and 0.98 Å (methyl H). Methyl group H atoms were allowed to rotate around the C—C bond to best fit the experimental electron density. *U*_iso_(H) values for all H atoms were constrained to a multiple of *U*_eq_ of their respective carrier atom (1.2 times for aromatic and carbonyl H atoms, and 1.5 times for methyl H atoms).

### Crystal data

**Table:**

[Mg_3_(C_8_H_4_O_4_)_3_(C_3_H_7_NO)_4_]	*F*(000) = 1792
*M**_r_* = 857.65	*D*_x_ = 1.396 Mg m^−^^3^
Monoclinic, *P*2_1_/*c*	Mo *K*α radiation, λ = 0.71073 Å
Hall symbol: -P 2ybc	Cell parameters from 4706 reflections
*a* = 18.158 (2) Å	θ = 2.3–30.2°
*b* = 9.5046 (13) Å	µ = 0.15 mm^−^^1^
*c* = 24.066 (3) Å	*T* = 100 K
β = 100.825 (2)°	Plate, colourless
*V* = 4079.6 (10) Å^3^	0.34 × 0.20 × 0.05 mm
*Z* = 4	

### Data collection

**Table:**

Bruker SMART APEX CCD diffractometer	10014 independent reflections
Radiation source: fine-focus sealed tube	7598 reflections with *I* > 2σ(*I*)
Graphite monochromator	*R*_int_ = 0.039
ω scans	θ_max_ = 28.3°, θ_min_ = 1.1°
Absorption correction: multi-scan (*SADABS*; Bruker, 2011)	*h* = −16→24
*T*_min_ = 0.619, *T*_max_ = 0.746	*k* = −12→12
21816 measured reflections	*l* = −32→27

### Refinement

**Table:**

Refinement on *F*^2^	Primary atom site location: structure-invariant direct methods
Least-squares matrix: full	Secondary atom site location: difference Fourier map
*R*[*F*^2^ > 2σ(*F*^2^)] = 0.066	Hydrogen site location: inferred from neighbouring sites
*wR*(*F*^2^) = 0.149	H-atom parameters constrained
*S* = 1.12	*w* = 1/[σ^2^(*F*_o_^2^) + (0.045*P*)^2^ + 5.8483*P*] where *P* = (*F*_o_^2^ + 2*F*_c_^2^)/3
10014 reflections	(Δ/σ)_max_ < 0.001
574 parameters	Δρ_max_ = 0.46 e Å^−^^3^
33 restraints	Δρ_min_ = −0.35 e Å^−^^3^

### Special details

**Table:**

Geometry. All e.s.d.'s (except the e.s.d. in the dihedral angle between two l.s. planes) are estimated using the full covariance matrix. The cell e.s.d.'s are taken into account individually in the estimation of e.s.d.'s in distances, angles and torsion angles; correlations between e.s.d.'s in cell parameters are only used when they are defined by crystal symmetry. An approximate (isotropic) treatment of cell e.s.d.'s is used for estimating e.s.d.'s involving l.s. planes.
Refinement. Refinement of *F*^2^ against ALL reflections. The weighted *R*-factor *wR* and goodness of fit *S* are based on *F*^2^, conventional *R*-factors *R* are based on *F*, with *F* set to zero for negative *F*^2^. The threshold expression of *F*^2^ > σ(*F*^2^) is used only for calculating *R*-factors(gt) *etc*. and is not relevant to the choice of reflections for refinement. *R*-factors based on *F*^2^ are statistically about twice as large as those based on *F*, and *R*- factors based on ALL data will be even larger.

### Fractional atomic coordinates and isotropic or equivalent isotropic displacement parameters (Å^2^)

**Table:**

	*x*	*y*	*z*	*U*_iso_*/*U*_eq_	Occ. (<1)
Mg1	0.20949 (5)	0.47092 (9)	0.09756 (4)	0.00978 (18)	
Mg2	0.25057 (4)	0.41016 (9)	0.24603 (4)	0.00810 (17)	
Mg3	0.28787 (5)	0.33064 (9)	0.39299 (4)	0.00983 (18)	
O1	0.30953 (10)	0.3705 (2)	0.11070 (8)	0.0152 (4)	
O2	0.33782 (10)	0.3615 (2)	0.20560 (8)	0.0146 (4)	
O3	0.67833 (10)	0.0492 (2)	0.20601 (8)	0.0138 (4)	
O4	0.64810 (10)	0.0028 (2)	0.11299 (8)	0.0147 (4)	
O5	0.14333 (10)	0.3020 (2)	0.10680 (8)	0.0138 (4)	
O6	0.17971 (10)	0.2685 (2)	0.20005 (8)	0.0132 (4)	
O7	−0.16247 (10)	−0.0372 (2)	0.21503 (8)	0.0135 (4)	
O8	−0.18736 (9)	−0.0683 (2)	0.12085 (8)	0.0134 (4)	
O9	0.26181 (10)	0.6786 (2)	0.12084 (8)	0.0140 (4)	
O10	0.21240 (9)	0.55376 (19)	0.18163 (7)	0.0104 (4)	
O11	0.23514 (10)	1.1286 (2)	0.36602 (8)	0.0135 (4)	
O12	0.28604 (9)	1.25894 (19)	0.30769 (7)	0.0106 (4)	
O13	0.21208 (10)	0.4826 (2)	0.01346 (8)	0.0175 (4)	
O14	0.10924 (10)	0.5856 (2)	0.07525 (8)	0.0169 (4)	
O16	0.28667 (10)	0.3291 (2)	0.47647 (8)	0.0160 (4)	
N1	0.26651 (13)	0.6147 (3)	−0.04601 (10)	0.0189 (5)	
N2	−0.01117 (12)	0.6279 (3)	0.08363 (11)	0.0204 (5)	
N4	0.24305 (14)	0.4812 (3)	0.53454 (11)	0.0203 (5)	
C1	0.35166 (14)	0.3411 (3)	0.15737 (12)	0.0128 (5)	
C2	0.42644 (14)	0.2735 (3)	0.15462 (12)	0.0143 (5)	
C3	0.44180 (14)	0.2137 (3)	0.10546 (12)	0.0158 (5)	
H3	0.4056	0.2187	0.0714	0.019*	
C4	0.51035 (14)	0.1461 (3)	0.10581 (11)	0.0152 (5)	
H4	0.5203	0.1042	0.0722	0.018*	
C5	0.56423 (14)	0.1400 (3)	0.15565 (11)	0.0135 (5)	
C6	0.54986 (16)	0.2045 (4)	0.20400 (13)	0.0272 (7)	
H6	0.5870	0.2041	0.2376	0.033*	
C7	0.48118 (16)	0.2702 (4)	0.20368 (13)	0.0280 (8)	
H7	0.4716	0.3132	0.2372	0.034*	
C8	0.63647 (13)	0.0582 (3)	0.15837 (11)	0.0116 (5)	
C9	0.13477 (13)	0.2544 (3)	0.15446 (11)	0.0112 (5)	
C10	0.06273 (13)	0.1758 (3)	0.15667 (11)	0.0115 (5)	
C11	0.01302 (14)	0.1379 (3)	0.10717 (11)	0.0141 (5)	
H11	0.0251	0.1584	0.0713	0.017*	
C12	−0.05394 (14)	0.0702 (3)	0.11033 (11)	0.0148 (5)	
H12	−0.0869	0.0430	0.0766	0.018*	
C13	−0.07311 (13)	0.0421 (3)	0.16273 (11)	0.0119 (5)	
C14	−0.02315 (15)	0.0784 (3)	0.21175 (12)	0.0196 (6)	
H14	−0.0352	0.0579	0.2476	0.024*	
C15	0.04437 (15)	0.1445 (3)	0.20874 (11)	0.0183 (6)	
H15	0.0781	0.1684	0.2426	0.022*	
C16	−0.14698 (13)	−0.0274 (3)	0.16669 (11)	0.0110 (5)	
C17	0.24114 (13)	0.6691 (3)	0.16770 (11)	0.0108 (5)	
C18	0.24572 (14)	0.7919 (3)	0.20711 (11)	0.0122 (5)	
C19	0.29401 (14)	0.9041 (3)	0.20265 (12)	0.0144 (5)	
H19	0.3251	0.9017	0.1750	0.017*	
C20	0.29669 (14)	1.0191 (3)	0.23864 (12)	0.0140 (5)	
H20	0.3299	1.0949	0.2359	0.017*	
C21	0.25055 (14)	1.0230 (3)	0.27868 (11)	0.0119 (5)	
C22	0.20191 (14)	0.9115 (3)	0.28294 (11)	0.0143 (5)	
H22	0.1704	0.9144	0.3103	0.017*	
C23	0.19968 (15)	0.7962 (3)	0.24713 (12)	0.0146 (5)	
H23	0.1666	0.7202	0.2500	0.018*	
C24	0.25574 (13)	1.1429 (3)	0.31948 (11)	0.0112 (5)	
C25	0.26467 (15)	0.5073 (3)	−0.01164 (11)	0.0149 (5)	
H25	0.3062	0.4447	−0.0056	0.018*	
C26	0.20341 (19)	0.7121 (4)	−0.05927 (15)	0.0313 (8)	
H26A	0.2223	0.8077	−0.0625	0.047*	
H26B	0.1740	0.7090	−0.0291	0.047*	
H26C	0.1716	0.6848	−0.0952	0.047*	
C27	0.32811 (18)	0.6340 (4)	−0.07655 (13)	0.0258 (7)	
H27A	0.3083	0.6350	−0.1173	0.039*	
H27B	0.3639	0.5565	−0.0675	0.039*	
H27C	0.3534	0.7234	−0.0653	0.039*	
C28	0.05722 (14)	0.5785 (3)	0.10208 (12)	0.0173 (6)	
H28	0.0672	0.5349	0.1382	0.021*	
C29	−0.03021 (19)	0.6938 (5)	0.02858 (15)	0.0386 (9)	
H29A	−0.0737	0.6460	0.0061	0.058*	
H29B	0.0125	0.6868	0.0092	0.058*	
H29C	−0.0423	0.7931	0.0332	0.058*	
C30	−0.07011 (17)	0.6193 (4)	0.11684 (15)	0.0325 (8)	
H30A	−0.1142	0.5730	0.0945	0.049*	
H30B	−0.0837	0.7143	0.1271	0.049*	
H30C	−0.0522	0.5648	0.1513	0.049*	
O15	0.39003 (12)	0.2178 (3)	0.41379 (13)	0.0163 (6)	0.923 (4)
C31	0.43687 (16)	0.2254 (4)	0.38262 (14)	0.0205 (7)	0.923 (4)
H31	0.4223	0.2736	0.3477	0.025*	0.923 (4)
N3	0.50494 (17)	0.1726 (3)	0.39358 (14)	0.0223 (7)	0.923 (4)
C32	0.55482 (19)	0.1786 (4)	0.35236 (17)	0.0331 (9)	0.923 (4)
H32A	0.6058	0.2014	0.3718	0.050*	0.923 (4)
H32B	0.5371	0.2512	0.3241	0.050*	0.923 (4)
H32C	0.5549	0.0871	0.3336	0.050*	0.923 (4)
C33	0.5317 (2)	0.0975 (6)	0.44580 (19)	0.0518 (14)	0.923 (4)
H33A	0.5822	0.1304	0.4624	0.078*	0.923 (4)
H33B	0.5332	−0.0035	0.4380	0.078*	0.923 (4)
H33C	0.4978	0.1149	0.4723	0.078*	0.923 (4)
O15B	0.3792 (13)	0.193 (4)	0.423 (2)	0.0163 (6)	0.077 (4)
C31B	0.4482 (14)	0.211 (4)	0.4268 (15)	0.021 (4)	0.077 (4)
H31B	0.4709	0.2819	0.4521	0.026*	0.077 (4)
N3B	0.4921 (15)	0.140 (4)	0.3991 (18)	0.0223 (7)	0.077 (4)
C32B	0.461 (2)	0.052 (5)	0.3498 (16)	0.034 (8)	0.077 (4)
H32D	0.4994	0.0368	0.3268	0.051*	0.077 (4)
H32E	0.4178	0.1004	0.3270	0.051*	0.077 (4)
H32F	0.4448	−0.0384	0.3627	0.051*	0.077 (4)
C33B	0.5715 (15)	0.168 (5)	0.405 (2)	0.041 (9)	0.077 (4)
H33D	0.5894	0.1304	0.3717	0.061*	0.077 (4)
H33E	0.5988	0.1230	0.4391	0.061*	0.077 (4)
H33F	0.5801	0.2698	0.4070	0.061*	0.077 (4)
C34	0.23760 (16)	0.3682 (3)	0.50274 (12)	0.0186 (6)	
H34	0.1934	0.3128	0.4996	0.022*	
C35	0.31082 (17)	0.5676 (4)	0.54180 (15)	0.0290 (7)	
H35A	0.2977	0.6662	0.5469	0.044*	
H35B	0.3338	0.5591	0.5082	0.044*	
H35C	0.3464	0.5355	0.5752	0.044*	
C36	0.18345 (18)	0.5209 (4)	0.56510 (14)	0.0275 (7)	
H36A	0.1673	0.6176	0.5551	0.041*	
H36B	0.2025	0.5149	0.6059	0.041*	
H36C	0.1408	0.4569	0.5547	0.041*	

### Atomic displacement parameters (Å^2^)

**Table:**

	*U*^11^	*U*^22^	*U*^33^	*U*^12^	*U*^13^	*U*^23^
Mg1	0.0078 (4)	0.0106 (4)	0.0116 (4)	0.0001 (3)	0.0037 (3)	−0.0002 (3)
Mg2	0.0061 (4)	0.0082 (4)	0.0103 (4)	0.0000 (3)	0.0024 (3)	−0.0007 (3)
Mg3	0.0090 (4)	0.0092 (4)	0.0117 (4)	−0.0002 (3)	0.0030 (3)	−0.0006 (3)
O1	0.0104 (9)	0.0187 (10)	0.0168 (10)	0.0035 (7)	0.0032 (7)	−0.0010 (8)
O2	0.0094 (8)	0.0197 (10)	0.0163 (10)	0.0038 (7)	0.0068 (7)	0.0006 (8)
O3	0.0107 (8)	0.0142 (10)	0.0159 (9)	0.0041 (7)	0.0007 (7)	0.0016 (8)
O4	0.0125 (9)	0.0167 (10)	0.0159 (10)	0.0066 (7)	0.0056 (7)	0.0014 (8)
O5	0.0131 (9)	0.0147 (9)	0.0142 (9)	−0.0053 (7)	0.0045 (7)	0.0003 (8)
O6	0.0109 (8)	0.0132 (9)	0.0146 (9)	−0.0035 (7)	−0.0002 (7)	−0.0018 (8)
O7	0.0110 (8)	0.0176 (10)	0.0135 (9)	−0.0025 (7)	0.0067 (7)	0.0001 (8)
O8	0.0097 (8)	0.0166 (10)	0.0140 (9)	−0.0026 (7)	0.0028 (7)	0.0007 (8)
O9	0.0168 (9)	0.0127 (9)	0.0142 (9)	−0.0011 (7)	0.0070 (7)	0.0001 (8)
O10	0.0111 (8)	0.0078 (8)	0.0122 (9)	0.0000 (6)	0.0017 (7)	0.0009 (7)
O11	0.0159 (9)	0.0117 (9)	0.0147 (9)	−0.0014 (7)	0.0075 (7)	−0.0018 (7)
O12	0.0101 (8)	0.0101 (9)	0.0119 (9)	−0.0008 (6)	0.0030 (7)	−0.0002 (7)
O13	0.0159 (9)	0.0250 (11)	0.0124 (9)	−0.0017 (8)	0.0049 (7)	−0.0014 (8)
O14	0.0121 (9)	0.0226 (11)	0.0166 (10)	0.0060 (8)	0.0046 (7)	0.0033 (8)
O16	0.0190 (9)	0.0188 (10)	0.0109 (9)	0.0029 (8)	0.0050 (7)	0.0003 (8)
N1	0.0228 (12)	0.0161 (12)	0.0194 (12)	−0.0002 (9)	0.0078 (10)	0.0002 (10)
N2	0.0111 (11)	0.0288 (14)	0.0217 (13)	0.0066 (10)	0.0042 (9)	0.0019 (11)
N4	0.0222 (12)	0.0177 (13)	0.0227 (13)	−0.0023 (9)	0.0082 (10)	−0.0024 (10)
C1	0.0094 (11)	0.0113 (12)	0.0185 (13)	−0.0007 (9)	0.0046 (10)	−0.0002 (10)
C2	0.0113 (12)	0.0156 (13)	0.0170 (13)	0.0032 (10)	0.0047 (10)	−0.0005 (11)
C3	0.0119 (12)	0.0212 (14)	0.0140 (13)	0.0043 (10)	0.0013 (10)	−0.0003 (11)
C4	0.0130 (12)	0.0201 (14)	0.0126 (13)	0.0070 (10)	0.0030 (10)	−0.0021 (11)
C5	0.0093 (11)	0.0155 (13)	0.0168 (13)	0.0025 (9)	0.0053 (10)	0.0010 (11)
C6	0.0144 (14)	0.048 (2)	0.0176 (15)	0.0133 (13)	−0.0013 (11)	−0.0077 (14)
C7	0.0175 (14)	0.051 (2)	0.0165 (14)	0.0158 (14)	0.0042 (11)	−0.0103 (14)
C8	0.0089 (11)	0.0093 (12)	0.0176 (13)	0.0001 (9)	0.0051 (9)	0.0026 (10)
C9	0.0080 (11)	0.0121 (12)	0.0142 (12)	−0.0002 (9)	0.0041 (9)	−0.0006 (10)
C10	0.0077 (11)	0.0134 (13)	0.0139 (13)	−0.0015 (9)	0.0033 (9)	0.0016 (10)
C11	0.0111 (12)	0.0203 (14)	0.0120 (12)	−0.0021 (10)	0.0051 (9)	0.0023 (11)
C12	0.0111 (12)	0.0194 (14)	0.0135 (13)	−0.0034 (10)	0.0013 (9)	0.0004 (11)
C13	0.0087 (11)	0.0157 (13)	0.0126 (12)	−0.0026 (9)	0.0054 (9)	0.0009 (10)
C14	0.0155 (13)	0.0323 (17)	0.0123 (13)	−0.0078 (12)	0.0055 (10)	0.0005 (12)
C15	0.0150 (13)	0.0284 (16)	0.0111 (13)	−0.0089 (11)	0.0014 (10)	−0.0006 (12)
C16	0.0095 (11)	0.0084 (12)	0.0159 (13)	0.0002 (9)	0.0048 (9)	0.0018 (10)
C17	0.0093 (11)	0.0093 (12)	0.0136 (12)	0.0022 (9)	0.0015 (9)	−0.0005 (10)
C18	0.0133 (12)	0.0084 (12)	0.0140 (13)	0.0005 (9)	0.0005 (10)	0.0008 (10)
C19	0.0144 (12)	0.0128 (13)	0.0177 (13)	−0.0016 (10)	0.0072 (10)	−0.0023 (11)
C20	0.0163 (12)	0.0079 (12)	0.0187 (13)	−0.0018 (9)	0.0055 (10)	0.0013 (10)
C21	0.0113 (11)	0.0096 (12)	0.0147 (13)	0.0022 (9)	0.0020 (9)	−0.0011 (10)
C22	0.0169 (12)	0.0110 (12)	0.0162 (13)	−0.0004 (10)	0.0067 (10)	−0.0005 (10)
C23	0.0164 (13)	0.0101 (12)	0.0186 (14)	−0.0026 (10)	0.0064 (10)	−0.0010 (10)
C24	0.0106 (11)	0.0100 (12)	0.0130 (12)	0.0007 (9)	0.0022 (9)	−0.0003 (10)
C25	0.0154 (12)	0.0154 (13)	0.0139 (13)	0.0003 (10)	0.0031 (10)	−0.0018 (10)
C26	0.0373 (19)	0.0222 (17)	0.0322 (18)	0.0086 (14)	0.0009 (14)	0.0043 (14)
C27	0.0329 (17)	0.0253 (17)	0.0223 (16)	−0.0101 (13)	0.0130 (13)	0.0015 (13)
C28	0.0141 (12)	0.0189 (14)	0.0190 (14)	0.0029 (10)	0.0032 (10)	0.0011 (11)
C29	0.0261 (17)	0.059 (3)	0.0301 (19)	0.0200 (17)	0.0031 (14)	0.0126 (18)
C30	0.0196 (15)	0.045 (2)	0.038 (2)	0.0080 (14)	0.0167 (14)	0.0013 (17)
O15	0.0123 (11)	0.0183 (14)	0.0193 (15)	0.0039 (9)	0.0054 (9)	0.0026 (9)
C31	0.0144 (14)	0.0218 (17)	0.0253 (17)	0.0065 (12)	0.0036 (12)	0.0014 (14)
N3	0.0122 (13)	0.0260 (18)	0.0293 (16)	0.0041 (11)	0.0056 (11)	0.0071 (14)
C32	0.0197 (17)	0.039 (2)	0.043 (2)	0.0097 (15)	0.0128 (16)	0.0039 (18)
C33	0.030 (2)	0.079 (4)	0.046 (3)	0.025 (2)	0.0068 (18)	0.030 (3)
O15B	0.0123 (11)	0.0183 (14)	0.0193 (15)	0.0039 (9)	0.0054 (9)	0.0026 (9)
C31B	0.015 (7)	0.024 (7)	0.026 (7)	0.007 (7)	0.005 (7)	0.006 (7)
N3B	0.0122 (13)	0.0260 (18)	0.0293 (16)	0.0041 (11)	0.0056 (11)	0.0071 (14)
C32B	0.021 (14)	0.037 (16)	0.049 (16)	−0.008 (13)	0.019 (13)	0.006 (15)
C33B	0.015 (14)	0.052 (17)	0.057 (17)	0.004 (14)	0.011 (14)	−0.013 (16)
C34	0.0201 (14)	0.0173 (14)	0.0188 (14)	−0.0015 (11)	0.0045 (11)	0.0010 (12)
C35	0.0259 (16)	0.0258 (17)	0.0372 (19)	−0.0053 (13)	0.0105 (14)	−0.0102 (15)
C36	0.0290 (17)	0.0271 (17)	0.0295 (17)	−0.0036 (13)	0.0138 (13)	−0.0079 (14)

### Geometric parameters (Å, º)

**Table:**

Mg1—O1	2.024 (2)	C10—C15	1.388 (4)
Mg1—O13	2.036 (2)	C10—C11	1.400 (4)
Mg1—O5	2.042 (2)	C11—C12	1.390 (4)
Mg1—O14	2.103 (2)	C11—H11	0.9500
Mg1—O10	2.162 (2)	C12—C13	1.396 (4)
Mg1—O9	2.217 (2)	C12—H12	0.9500
Mg1—C17	2.522 (3)	C13—C14	1.390 (4)
Mg1—Mg2	3.5575 (13)	C13—C16	1.514 (3)
Mg2—O6	2.0393 (19)	C14—C15	1.392 (4)
Mg2—O3^i^	2.046 (2)	C14—H14	0.9500
Mg2—O2	2.0614 (19)	C15—H15	0.9500
Mg2—O7^ii^	2.0619 (19)	C17—C18	1.497 (4)
Mg2—O12^iii^	2.079 (2)	C18—C23	1.390 (4)
Mg2—O10	2.0837 (19)	C18—C19	1.397 (4)
Mg2—Mg3	3.5550 (13)	C19—C20	1.390 (4)
Mg3—O16	2.013 (2)	C19—H19	0.9500
Mg3—O4^i^	2.028 (2)	C20—C21	1.391 (4)
Mg3—O8^ii^	2.0338 (19)	C20—H20	0.9500
Mg3—O15	2.120 (2)	C21—C22	1.396 (4)
Mg3—O15B	2.127 (18)	C21—C24	1.496 (4)
Mg3—O12^iii^	2.157 (2)	C22—C23	1.390 (4)
Mg3—O11^iii^	2.189 (2)	C22—H22	0.9500
Mg3—C24^iii^	2.503 (3)	C23—H23	0.9500
O1—C1	1.266 (3)	C24—Mg3^vi^	2.503 (3)
O2—C1	1.248 (3)	C25—H25	0.9500
O3—C8	1.254 (3)	C26—H26A	0.9800
O3—Mg2^iv^	2.0456 (19)	C26—H26B	0.9800
O4—C8	1.265 (3)	C26—H26C	0.9800
O4—Mg3^iv^	2.028 (2)	C27—H27A	0.9800
O5—C9	1.270 (3)	C27—H27B	0.9800
O6—C9	1.246 (3)	C27—H27C	0.9800
O7—C16	1.251 (3)	C28—H28	0.9500
O7—Mg2^v^	2.0618 (19)	C29—H29A	0.9800
O8—C16	1.266 (3)	C29—H29B	0.9800
O8—Mg3^v^	2.0338 (19)	C29—H29C	0.9800
O9—C17	1.257 (3)	C30—H30A	0.9800
O10—C17	1.285 (3)	C30—H30B	0.9800
O11—C24	1.253 (3)	C30—H30C	0.9800
O11—Mg3^vi^	2.189 (2)	O15—C31	1.237 (4)
O12—C24	1.288 (3)	C31—N3	1.314 (4)
O12—Mg2^vi^	2.079 (2)	C31—H31	0.9500
O12—Mg3^vi^	2.157 (2)	N3—C33	1.448 (5)
O13—C25	1.244 (3)	N3—C32	1.465 (4)
O14—C28	1.242 (3)	C32—H32A	0.9800
O16—C34	1.242 (3)	C32—H32B	0.9800
N1—C25	1.318 (4)	C32—H32C	0.9800
N1—C26	1.461 (4)	C33—H33A	0.9800
N1—C27	1.461 (4)	C33—H33B	0.9800
N2—C28	1.324 (3)	C33—H33C	0.9800
N2—C29	1.447 (4)	O15B—C31B	1.250 (19)
N2—C30	1.453 (4)	C31B—N3B	1.318 (18)
N4—C34	1.312 (4)	C31B—H31B	0.9500
N4—C35	1.463 (4)	N3B—C33B	1.448 (18)
N4—C36	1.468 (4)	N3B—C32B	1.473 (19)
C1—C2	1.515 (3)	C32B—H32D	0.9800
C2—C3	1.388 (4)	C32B—H32E	0.9800
C2—C7	1.394 (4)	C32B—H32F	0.9800
C3—C4	1.399 (4)	C33B—H33D	0.9800
C3—H3	0.9500	C33B—H33E	0.9800
C4—C5	1.399 (4)	C33B—H33F	0.9800
C4—H4	0.9500	C34—H34	0.9500
C5—C6	1.383 (4)	C35—H35A	0.9800
C5—C8	1.516 (3)	C35—H35B	0.9800
C6—C7	1.393 (4)	C35—H35C	0.9800
C6—H6	0.9500	C36—H36A	0.9800
C7—H7	0.9500	C36—H36B	0.9800
C9—C10	1.516 (3)	C36—H36C	0.9800
			
O1—Mg1—O13	89.62 (8)	C5—C6—C7	120.3 (3)
O1—Mg1—O5	98.14 (9)	C5—C6—H6	119.9
O13—Mg1—O5	105.97 (9)	C7—C6—H6	119.9
O1—Mg1—O14	173.16 (9)	C6—C7—C2	120.7 (3)
O13—Mg1—O14	84.17 (8)	C6—C7—H7	119.7
O5—Mg1—O14	86.35 (8)	C2—C7—H7	119.7
O1—Mg1—O10	99.32 (8)	O3—C8—O4	126.1 (2)
O13—Mg1—O10	155.37 (9)	O3—C8—C5	116.5 (2)
O5—Mg1—O10	95.50 (8)	O4—C8—C5	117.4 (2)
O14—Mg1—O10	85.30 (8)	O6—C9—O5	125.9 (2)
O1—Mg1—O9	92.91 (8)	O6—C9—C10	116.7 (2)
O13—Mg1—O9	96.53 (8)	O5—C9—C10	117.4 (2)
O5—Mg1—O9	154.90 (8)	C15—C10—C11	119.2 (2)
O14—Mg1—O9	84.98 (8)	C15—C10—C9	119.5 (2)
O10—Mg1—O9	60.35 (7)	C11—C10—C9	121.3 (2)
O1—Mg1—C17	99.35 (8)	C12—C11—C10	120.2 (2)
O13—Mg1—C17	125.41 (9)	C12—C11—H11	119.9
O5—Mg1—C17	125.37 (9)	C10—C11—H11	119.9
O14—Mg1—C17	82.04 (8)	C11—C12—C13	120.5 (2)
O10—Mg1—C17	30.63 (7)	C11—C12—H12	119.8
O9—Mg1—C17	29.88 (8)	C13—C12—H12	119.8
O1—Mg1—Mg2	75.75 (6)	C14—C13—C12	119.0 (2)
O13—Mg1—Mg2	165.35 (7)	C14—C13—C16	120.0 (2)
O5—Mg1—Mg2	77.27 (6)	C12—C13—C16	121.0 (2)
O14—Mg1—Mg2	110.41 (6)	C13—C14—C15	120.6 (3)
O10—Mg1—Mg2	32.40 (5)	C13—C14—H14	119.7
O9—Mg1—Mg2	83.78 (6)	C15—C14—H14	119.7
C17—Mg1—Mg2	58.04 (6)	C10—C15—C14	120.5 (2)
O6—Mg2—O3^i^	178.51 (9)	C10—C15—H15	119.8
O6—Mg2—O2	93.34 (8)	C14—C15—H15	119.8
O3^i^—Mg2—O2	87.23 (8)	O7—C16—O8	126.4 (2)
O6—Mg2—O7^ii^	86.70 (8)	O7—C16—C13	116.6 (2)
O3^i^—Mg2—O7^ii^	92.76 (8)	O8—C16—C13	117.0 (2)
O2—Mg2—O7^ii^	178.54 (9)	O9—C17—O10	120.0 (2)
O6—Mg2—O12^iii^	90.58 (8)	O9—C17—C18	121.5 (2)
O3^i^—Mg2—O12^iii^	88.04 (8)	O10—C17—C18	118.5 (2)
O2—Mg2—O12^iii^	91.11 (8)	O9—C17—Mg1	61.46 (14)
O7^ii^—Mg2—O12^iii^	90.35 (8)	O10—C17—Mg1	59.02 (13)
O6—Mg2—O10	86.62 (8)	C18—C17—Mg1	170.10 (17)
O3^i^—Mg2—O10	94.77 (8)	C23—C18—C19	119.9 (2)
O2—Mg2—O10	88.49 (8)	C23—C18—C17	119.5 (2)
O7^ii^—Mg2—O10	90.06 (8)	C19—C18—C17	120.5 (2)
O12^iii^—Mg2—O10	177.14 (8)	C20—C19—C18	120.1 (2)
O6—Mg2—Mg3	112.48 (6)	C20—C19—H19	120.0
O3^i^—Mg2—Mg3	66.04 (6)	C18—C19—H19	120.0
O2—Mg2—Mg3	113.72 (6)	C19—C20—C21	119.8 (2)
O7^ii^—Mg2—Mg3	67.58 (6)	C19—C20—H20	120.1
O12^iii^—Mg2—Mg3	33.64 (5)	C21—C20—H20	120.1
O10—Mg2—Mg3	148.63 (6)	C20—C21—C22	120.2 (2)
O6—Mg2—Mg1	64.55 (6)	C20—C21—C24	120.2 (2)
O3^i^—Mg2—Mg1	116.93 (6)	C22—C21—C24	119.5 (2)
O2—Mg2—Mg1	66.24 (6)	C23—C22—C21	119.9 (2)
O7^ii^—Mg2—Mg1	112.51 (6)	C23—C22—H22	120.1
O12^iii^—Mg2—Mg1	143.84 (6)	C21—C22—H22	120.1
O10—Mg2—Mg1	33.79 (5)	C18—C23—C22	120.1 (2)
Mg3—Mg2—Mg1	176.90 (3)	C18—C23—H23	120.0
O16—Mg3—O4^i^	100.97 (9)	C22—C23—H23	120.0
O16—Mg3—O8^ii^	89.38 (8)	O11—C24—O12	120.1 (2)
O4^i^—Mg3—O8^ii^	96.67 (9)	O11—C24—C21	120.8 (2)
O16—Mg3—O15	86.20 (10)	O12—C24—C21	119.0 (2)
O4^i^—Mg3—O15	86.40 (10)	O11—C24—Mg3^vi^	60.98 (14)
O8^ii^—Mg3—O15	175.03 (10)	O12—C24—Mg3^vi^	59.53 (13)
O16—Mg3—O15B	79.4 (12)	C21—C24—Mg3^vi^	169.95 (18)
O4^i^—Mg3—O15B	95.7 (10)	O13—C25—N1	124.3 (3)
O8^ii^—Mg3—O15B	164.7 (9)	O13—C25—H25	117.9
O15—Mg3—O15B	10.6 (9)	N1—C25—H25	117.9
O16—Mg3—O12^iii^	161.12 (9)	N1—C26—H26A	109.5
O4^i^—Mg3—O12^iii^	95.39 (8)	N1—C26—H26B	109.5
O8^ii^—Mg3—O12^iii^	98.07 (8)	H26A—C26—H26B	109.5
O15—Mg3—O12^iii^	85.49 (11)	N1—C26—H26C	109.5
O15B—Mg3—O12^iii^	89.8 (13)	H26A—C26—H26C	109.5
O16—Mg3—O11^iii^	101.73 (8)	H26B—C26—H26C	109.5
O4^i^—Mg3—O11^iii^	155.71 (9)	N1—C27—H27A	109.5
O8^ii^—Mg3—O11^iii^	92.10 (8)	N1—C27—H27B	109.5
O15—Mg3—O11^iii^	86.60 (10)	H27A—C27—H27B	109.5
O15B—Mg3—O11^iii^	80.2 (12)	N1—C27—H27C	109.5
O12^iii^—Mg3—O11^iii^	60.87 (7)	H27A—C27—H27C	109.5
O16—Mg3—C24^iii^	130.95 (9)	H27B—C27—H27C	109.5
O4^i^—Mg3—C24^iii^	125.85 (9)	O14—C28—N2	124.3 (3)
O8^ii^—Mg3—C24^iii^	97.93 (8)	O14—C28—H28	117.8
O15—Mg3—C24^iii^	83.32 (11)	N2—C28—H28	117.8
O15B—Mg3—C24^iii^	82.0 (14)	N2—C29—H29A	109.5
O12^iii^—Mg3—C24^iii^	30.96 (8)	N2—C29—H29B	109.5
O11^iii^—Mg3—C24^iii^	30.04 (8)	H29A—C29—H29B	109.5
O16—Mg3—Mg2	163.43 (7)	N2—C29—H29C	109.5
O4^i^—Mg3—Mg2	76.26 (6)	H29A—C29—H29C	109.5
O8^ii^—Mg3—Mg2	74.89 (6)	H29B—C29—H29C	109.5
O15—Mg3—Mg2	109.72 (9)	N2—C30—H30A	109.5
O15B—Mg3—Mg2	117.1 (12)	N2—C30—H30B	109.5
O12^iii^—Mg3—Mg2	32.27 (5)	H30A—C30—H30B	109.5
O11^iii^—Mg3—Mg2	84.30 (6)	N2—C30—H30C	109.5
C24^iii^—Mg3—Mg2	58.19 (6)	H30A—C30—H30C	109.5
C1—O1—Mg1	128.18 (18)	H30B—C30—H30C	109.5
C1—O2—Mg2	141.34 (17)	C31—O15—Mg3	119.9 (2)
C8—O3—Mg2^iv^	140.36 (18)	O15—C31—N3	125.7 (3)
C8—O4—Mg3^iv^	126.01 (17)	O15—C31—H31	117.1
C9—O5—Mg1	123.60 (17)	N3—C31—H31	117.1
C9—O6—Mg2	142.32 (18)	C31—N3—C33	120.8 (3)
C16—O7—Mg2^v^	140.37 (17)	C31—N3—C32	121.8 (3)
C16—O8—Mg3^v^	130.02 (17)	C33—N3—C32	117.2 (3)
C17—O9—Mg1	88.66 (15)	C31B—O15B—Mg3	130 (3)
C17—O10—Mg2	131.15 (16)	O15B—C31B—N3B	126 (3)
C17—O10—Mg1	90.35 (15)	O15B—C31B—H31B	117.2
Mg2—O10—Mg1	113.81 (9)	N3B—C31B—H31B	117.2
C24—O11—Mg3^vi^	88.98 (15)	C31B—N3B—C33B	123 (2)
C24—O12—Mg2^vi^	131.40 (16)	C31B—N3B—C32B	121 (2)
C24—O12—Mg3^vi^	89.52 (15)	C33B—N3B—C32B	114 (2)
Mg2^vi^—O12—Mg3^vi^	114.08 (9)	N3B—C32B—H32D	109.5
C25—O13—Mg1	130.98 (18)	N3B—C32B—H32E	109.5
C28—O14—Mg1	123.08 (18)	H32D—C32B—H32E	109.5
C34—O16—Mg3	130.47 (19)	N3B—C32B—H32F	109.5
C25—N1—C26	121.1 (3)	H32D—C32B—H32F	109.5
C25—N1—C27	121.7 (3)	H32E—C32B—H32F	109.5
C26—N1—C27	117.0 (3)	N3B—C33B—H33D	109.5
C28—N2—C29	120.5 (3)	N3B—C33B—H33E	109.5
C28—N2—C30	122.5 (3)	H33D—C33B—H33E	109.5
C29—N2—C30	117.0 (2)	N3B—C33B—H33F	109.5
C34—N4—C35	120.0 (3)	H33D—C33B—H33F	109.5
C34—N4—C36	121.4 (3)	H33E—C33B—H33F	109.5
C35—N4—C36	118.6 (3)	O16—C34—N4	124.0 (3)
O2—C1—O1	126.7 (2)	O16—C34—H34	118.0
O2—C1—C2	116.5 (2)	N4—C34—H34	118.0
O1—C1—C2	116.9 (2)	N4—C35—H35A	109.5
C3—C2—C7	119.2 (2)	N4—C35—H35B	109.5
C3—C2—C1	122.4 (2)	H35A—C35—H35B	109.5
C7—C2—C1	118.4 (2)	N4—C35—H35C	109.5
C2—C3—C4	120.3 (2)	H35A—C35—H35C	109.5
C2—C3—H3	119.9	H35B—C35—H35C	109.5
C4—C3—H3	119.9	N4—C36—H36A	109.5
C5—C4—C3	120.1 (2)	N4—C36—H36B	109.5
C5—C4—H4	119.9	H36A—C36—H36B	109.5
C3—C4—H4	119.9	N4—C36—H36C	109.5
C6—C5—C4	119.4 (2)	H36A—C36—H36C	109.5
C6—C5—C8	119.3 (2)	H36B—C36—H36C	109.5
C4—C5—C8	121.2 (2)		
			
O1—Mg1—Mg2—O6	−98.15 (9)	O9—Mg1—O13—C25	53.0 (3)
O13—Mg1—Mg2—O6	−100.7 (3)	C17—Mg1—O13—C25	61.3 (3)
O5—Mg1—Mg2—O6	3.81 (8)	Mg2—Mg1—O13—C25	−37.4 (5)
O14—Mg1—Mg2—O6	84.97 (9)	O13—Mg1—O14—C28	152.1 (2)
O10—Mg1—Mg2—O6	126.44 (11)	O5—Mg1—O14—C28	45.6 (2)
O9—Mg1—Mg2—O6	167.24 (8)	O10—Mg1—O14—C28	−50.2 (2)
C17—Mg1—Mg2—O6	150.99 (9)	O9—Mg1—O14—C28	−110.8 (2)
O1—Mg1—Mg2—O3^i^	81.68 (9)	C17—Mg1—O14—C28	−80.8 (2)
O13—Mg1—Mg2—O3^i^	79.1 (3)	Mg2—Mg1—O14—C28	−29.3 (2)
O5—Mg1—Mg2—O3^i^	−176.36 (9)	O4^i^—Mg3—O16—C34	101.4 (3)
O14—Mg1—Mg2—O3^i^	−95.20 (9)	O8^ii^—Mg3—O16—C34	4.7 (3)
O10—Mg1—Mg2—O3^i^	−53.73 (11)	O15—Mg3—O16—C34	−173.0 (3)
O9—Mg1—Mg2—O3^i^	−12.93 (9)	O15B—Mg3—O16—C34	−164.8 (11)
C17—Mg1—Mg2—O3^i^	−29.18 (9)	O12^iii^—Mg3—O16—C34	−109.0 (3)
O1—Mg1—Mg2—O2	8.12 (9)	O11^iii^—Mg3—O16—C34	−87.3 (3)
O13—Mg1—Mg2—O2	5.5 (3)	C24^iii^—Mg3—O16—C34	−95.3 (3)
O5—Mg1—Mg2—O2	110.07 (9)	Mg2—Mg3—O16—C34	22.7 (4)
O14—Mg1—Mg2—O2	−168.77 (9)	Mg2—O2—C1—O1	13.8 (5)
O10—Mg1—Mg2—O2	−127.30 (11)	Mg2—O2—C1—C2	−165.9 (2)
O9—Mg1—Mg2—O2	−86.50 (8)	Mg1—O1—C1—O2	4.3 (4)
C17—Mg1—Mg2—O2	−102.74 (9)	Mg1—O1—C1—C2	−175.96 (17)
O1—Mg1—Mg2—O7^ii^	−172.71 (9)	O2—C1—C2—C3	163.9 (3)
O13—Mg1—Mg2—O7^ii^	−175.3 (3)	O1—C1—C2—C3	−15.9 (4)
O5—Mg1—Mg2—O7^ii^	−70.76 (9)	O2—C1—C2—C7	−15.3 (4)
O14—Mg1—Mg2—O7^ii^	10.41 (10)	O1—C1—C2—C7	164.9 (3)
O10—Mg1—Mg2—O7^ii^	51.87 (11)	C7—C2—C3—C4	2.5 (4)
O9—Mg1—Mg2—O7^ii^	92.68 (8)	C1—C2—C3—C4	−176.7 (3)
C17—Mg1—Mg2—O7^ii^	76.43 (9)	C2—C3—C4—C5	−0.8 (4)
O1—Mg1—Mg2—O12^iii^	−47.37 (12)	C3—C4—C5—C6	−1.7 (4)
O13—Mg1—Mg2—O12^iii^	−50.0 (3)	C3—C4—C5—C8	175.4 (3)
O5—Mg1—Mg2—O12^iii^	54.58 (11)	C4—C5—C6—C7	2.5 (5)
O14—Mg1—Mg2—O12^iii^	135.75 (11)	C8—C5—C6—C7	−174.6 (3)
O10—Mg1—Mg2—O12^iii^	177.21 (14)	C5—C6—C7—C2	−0.8 (6)
O9—Mg1—Mg2—O12^iii^	−141.99 (11)	C3—C2—C7—C6	−1.7 (5)
C17—Mg1—Mg2—O12^iii^	−158.23 (12)	C1—C2—C7—C6	177.5 (3)
O1—Mg1—Mg2—O10	135.42 (11)	Mg2^iv^—O3—C8—O4	−34.2 (4)
O13—Mg1—Mg2—O10	132.8 (3)	Mg2^iv^—O3—C8—C5	144.0 (2)
O5—Mg1—Mg2—O10	−122.63 (11)	Mg3^iv^—O4—C8—O3	24.5 (4)
O14—Mg1—Mg2—O10	−41.47 (11)	Mg3^iv^—O4—C8—C5	−153.63 (18)
O9—Mg1—Mg2—O10	40.80 (10)	C6—C5—C8—O3	2.9 (4)
C17—Mg1—Mg2—O10	24.56 (11)	C4—C5—C8—O3	−174.2 (3)
O6—Mg2—Mg3—O16	−100.1 (2)	C6—C5—C8—O4	−178.8 (3)
O3^i^—Mg2—Mg3—O16	80.0 (2)	C4—C5—C8—O4	4.1 (4)
O2—Mg2—Mg3—O16	155.4 (2)	Mg2—O6—C9—O5	39.7 (5)
O7^ii^—Mg2—Mg3—O16	−23.9 (2)	Mg2—O6—C9—C10	−138.9 (2)
O12^iii^—Mg2—Mg3—O16	−153.1 (3)	Mg1—O5—C9—O6	−24.4 (4)
O10—Mg2—Mg3—O16	23.7 (3)	Mg1—O5—C9—C10	154.17 (18)
O6—Mg2—Mg3—O4^i^	177.61 (9)	O6—C9—C10—C15	10.5 (4)
O3^i^—Mg2—Mg3—O4^i^	−2.25 (8)	O5—C9—C10—C15	−168.1 (3)
O2—Mg2—Mg3—O4^i^	73.09 (9)	O6—C9—C10—C11	−171.5 (2)
O7^ii^—Mg2—Mg3—O4^i^	−106.16 (9)	O5—C9—C10—C11	9.8 (4)
O12^iii^—Mg2—Mg3—O4^i^	124.63 (11)	C15—C10—C11—C12	0.2 (4)
O10—Mg2—Mg3—O4^i^	−58.60 (13)	C9—C10—C11—C12	−177.7 (3)
O6—Mg2—Mg3—O8^ii^	−81.45 (9)	C10—C11—C12—C13	1.4 (4)
O3^i^—Mg2—Mg3—O8^ii^	98.69 (9)	C11—C12—C13—C14	−2.1 (4)
O2—Mg2—Mg3—O8^ii^	174.04 (9)	C11—C12—C13—C16	178.1 (3)
O7^ii^—Mg2—Mg3—O8^ii^	−5.22 (8)	C12—C13—C14—C15	1.3 (5)
O12^iii^—Mg2—Mg3—O8^ii^	−134.43 (11)	C16—C13—C14—C15	−178.9 (3)
O10—Mg2—Mg3—O8^ii^	42.34 (13)	C11—C10—C15—C14	−1.0 (4)
O6—Mg2—Mg3—O15	96.60 (11)	C9—C10—C15—C14	177.0 (3)
O3^i^—Mg2—Mg3—O15	−83.26 (11)	C13—C14—C15—C10	0.3 (5)
O2—Mg2—Mg3—O15	−7.92 (12)	Mg2^v^—O7—C16—O8	−9.1 (5)
O7^ii^—Mg2—Mg3—O15	172.82 (11)	Mg2^v^—O7—C16—C13	170.08 (19)
O12^iii^—Mg2—Mg3—O15	43.62 (12)	Mg3^v^—O8—C16—O7	−2.5 (4)
O10—Mg2—Mg3—O15	−139.61 (14)	Mg3^v^—O8—C16—C13	178.32 (17)
O6—Mg2—Mg3—O15B	88.2 (12)	C14—C13—C16—O7	6.4 (4)
O3^i^—Mg2—Mg3—O15B	−91.7 (12)	C12—C13—C16—O7	−173.8 (2)
O2—Mg2—Mg3—O15B	−16.3 (12)	C14—C13—C16—O8	−174.4 (3)
O7^ii^—Mg2—Mg3—O15B	164.4 (12)	C12—C13—C16—O8	5.4 (4)
O12^iii^—Mg2—Mg3—O15B	35.2 (12)	Mg1—O9—C17—O10	7.9 (2)
O10—Mg2—Mg3—O15B	−148.0 (12)	Mg1—O9—C17—C18	−169.1 (2)
O6—Mg2—Mg3—O12^iii^	52.98 (11)	Mg2—O10—C17—O9	114.6 (2)
O3^i^—Mg2—Mg3—O12^iii^	−126.88 (11)	Mg1—O10—C17—O9	−8.1 (2)
O2—Mg2—Mg3—O12^iii^	−51.54 (11)	Mg2—O10—C17—C18	−68.3 (3)
O7^ii^—Mg2—Mg3—O12^iii^	129.21 (11)	Mg1—O10—C17—C18	168.96 (19)
O10—Mg2—Mg3—O12^iii^	176.77 (15)	Mg2—O10—C17—Mg1	122.78 (19)
O6—Mg2—Mg3—O11^iii^	12.28 (8)	O1—Mg1—C17—O9	79.45 (15)
O3^i^—Mg2—Mg3—O11^iii^	−167.58 (8)	O13—Mg1—C17—O9	−16.80 (18)
O2—Mg2—Mg3—O11^iii^	−92.23 (9)	O5—Mg1—C17—O9	−173.53 (14)
O7^ii^—Mg2—Mg3—O11^iii^	88.51 (8)	O14—Mg1—C17—O9	−93.78 (15)
O12^iii^—Mg2—Mg3—O11^iii^	−40.70 (10)	O10—Mg1—C17—O9	172.0 (2)
O10—Mg2—Mg3—O11^iii^	136.07 (12)	Mg2—Mg1—C17—O9	146.06 (16)
O6—Mg2—Mg3—C24^iii^	28.17 (9)	O1—Mg1—C17—O10	−92.53 (14)
O3^i^—Mg2—Mg3—C24^iii^	−151.69 (9)	O13—Mg1—C17—O10	171.22 (13)
O2—Mg2—Mg3—C24^iii^	−76.35 (10)	O5—Mg1—C17—O10	14.49 (17)
O7^ii^—Mg2—Mg3—C24^iii^	104.40 (9)	O14—Mg1—C17—O10	94.24 (14)
O12^iii^—Mg2—Mg3—C24^iii^	−24.81 (11)	O9—Mg1—C17—O10	−172.0 (2)
O10—Mg2—Mg3—C24^iii^	151.96 (13)	Mg2—Mg1—C17—O10	−25.92 (11)
O13—Mg1—O1—C1	170.4 (2)	O9—C17—C18—C23	156.9 (2)
O5—Mg1—O1—C1	−83.5 (2)	O10—C17—C18—C23	−20.2 (3)
O10—Mg1—O1—C1	13.5 (2)	O9—C17—C18—C19	−21.0 (4)
O9—Mg1—O1—C1	73.9 (2)	O10—C17—C18—C19	162.0 (2)
C17—Mg1—O1—C1	44.5 (2)	C23—C18—C19—C20	0.8 (4)
Mg2—Mg1—O1—C1	−8.9 (2)	C17—C18—C19—C20	178.6 (2)
O6—Mg2—O2—C1	45.6 (3)	C18—C19—C20—C21	−0.7 (4)
O3^i^—Mg2—O2—C1	−135.8 (3)	C19—C20—C21—C22	0.3 (4)
O12^iii^—Mg2—O2—C1	136.3 (3)	C19—C20—C21—C24	177.0 (2)
O10—Mg2—O2—C1	−40.9 (3)	C20—C21—C22—C23	0.1 (4)
Mg1—Mg2—O2—C1	−14.6 (3)	C24—C21—C22—C23	−176.6 (2)
O1—Mg1—O5—C9	81.0 (2)	C19—C18—C23—C22	−0.4 (4)
O13—Mg1—O5—C9	172.94 (19)	C17—C18—C23—C22	−178.2 (2)
O14—Mg1—O5—C9	−104.2 (2)	C21—C22—C23—C18	−0.1 (4)
O10—Mg1—O5—C9	−19.3 (2)	Mg3^vi^—O11—C24—O12	−7.1 (2)
O9—Mg1—O5—C9	−34.2 (3)	Mg3^vi^—O11—C24—C21	168.6 (2)
C17—Mg1—O5—C9	−26.6 (2)	Mg2^vi^—O12—C24—O11	−115.2 (2)
Mg2—Mg1—O5—C9	7.69 (18)	Mg3^vi^—O12—C24—O11	7.2 (2)
O2—Mg2—O6—C9	−85.1 (3)	Mg2^vi^—O12—C24—C21	69.0 (3)
O7^ii^—Mg2—O6—C9	93.4 (3)	Mg3^vi^—O12—C24—C21	−168.5 (2)
O12^iii^—Mg2—O6—C9	−176.3 (3)	Mg2^vi^—O12—C24—Mg3^vi^	−122.45 (19)
O10—Mg2—O6—C9	3.1 (3)	C20—C21—C24—O11	−156.0 (2)
Mg3—Mg2—O6—C9	157.5 (3)	C22—C21—C24—O11	20.7 (4)
Mg1—Mg2—O6—C9	−23.5 (3)	C20—C21—C24—O12	19.7 (4)
O1—Mg1—O9—C17	−103.77 (15)	C22—C21—C24—O12	−163.6 (2)
O13—Mg1—O9—C17	166.29 (15)	C20—C21—C24—Mg3^vi^	−59.0 (12)
O5—Mg1—O9—C17	12.5 (3)	C22—C21—C24—Mg3^vi^	117.7 (10)
O14—Mg1—O9—C17	82.75 (15)	Mg1—O13—C25—N1	−119.3 (3)
O10—Mg1—O9—C17	−4.69 (13)	C26—N1—C25—O13	−2.7 (4)
Mg2—Mg1—O9—C17	−28.45 (13)	C27—N1—C25—O13	−176.8 (3)
O6—Mg2—O10—C17	−159.9 (2)	Mg1—O14—C28—N2	−166.9 (2)
O3^i^—Mg2—O10—C17	20.6 (2)	C29—N2—C28—O14	0.9 (5)
O2—Mg2—O10—C17	−66.5 (2)	C30—N2—C28—O14	−179.0 (3)
O7^ii^—Mg2—O10—C17	113.4 (2)	O16—Mg3—O15—C31	−153.7 (3)
Mg3—Mg2—O10—C17	70.4 (3)	O4^i^—Mg3—O15—C31	−52.5 (3)
Mg1—Mg2—O10—C17	−113.2 (2)	O15B—Mg3—O15—C31	157 (8)
O6—Mg2—O10—Mg1	−46.69 (9)	O12^iii^—Mg3—O15—C31	43.2 (3)
O3^i^—Mg2—O10—Mg1	133.84 (9)	O11^iii^—Mg3—O15—C31	104.3 (3)
O2—Mg2—O10—Mg1	46.74 (10)	C24^iii^—Mg3—O15—C31	74.3 (3)
O7^ii^—Mg2—O10—Mg1	−133.39 (9)	Mg2—Mg3—O15—C31	21.6 (3)
Mg3—Mg2—O10—Mg1	−176.40 (6)	Mg3—O15—C31—N3	172.7 (3)
O1—Mg1—O10—C17	92.63 (14)	O15—C31—N3—C33	0.0 (6)
O13—Mg1—O10—C17	−17.4 (3)	O15—C31—N3—C32	175.8 (4)
O5—Mg1—O10—C17	−168.17 (14)	O16—Mg3—O15B—C31B	−109 (4)
O14—Mg1—O10—C17	−82.30 (14)	O4^i^—Mg3—O15B—C31B	−9 (4)
O9—Mg1—O10—C17	4.59 (13)	O8^ii^—Mg3—O15B—C31B	−152 (3)
Mg2—Mg1—O10—C17	136.21 (17)	O15—Mg3—O15B—C31B	21 (5)
O1—Mg1—O10—Mg2	−43.59 (10)	O12^iii^—Mg3—O15B—C31B	87 (4)
O13—Mg1—O10—Mg2	−153.57 (16)	O11^iii^—Mg3—O15B—C31B	147 (5)
O5—Mg1—O10—Mg2	55.62 (10)	C24^iii^—Mg3—O15B—C31B	117 (4)
O14—Mg1—O10—Mg2	141.49 (10)	Mg2—Mg3—O15B—C31B	69 (5)
O9—Mg1—O10—Mg2	−131.63 (11)	Mg3—O15B—C31B—N3B	−117 (5)
C17—Mg1—O10—Mg2	−136.21 (17)	O15B—C31B—N3B—C33B	178 (5)
O1—Mg1—O13—C25	−39.9 (3)	O15B—C31B—N3B—C32B	15 (7)
O5—Mg1—O13—C25	−138.3 (2)	Mg3—O16—C34—N4	−108.5 (3)
O14—Mg1—O13—C25	137.2 (3)	C35—N4—C34—O16	−1.6 (5)
O10—Mg1—O13—C25	72.1 (3)	C36—N4—C34—O16	−179.3 (3)

Symmetry codes: (i) −*x*+1, *y*+1/2, −*z*+1/2; (ii) −*x*, *y*+1/2, −*z*+1/2; (iii) *x*, *y*−1, *z*; (iv) −*x*+1, *y*−1/2, −*z*+1/2; (v) −*x*, *y*−1/2, −*z*+1/2; (vi) *x*, *y*+1, *z*.
